# Probable Spinal Neuroschistosomiasis Manifesting as Transverse Myelitis

**DOI:** 10.4269/ajtmh.21-0649

**Published:** 2021-08-09

**Authors:** Rusheng Chew

**Affiliations:** Mahidol Oxford Tropical Medicine Research Unit, 420/6 Ratchawithi Road, Ratchathewi, Bangkok 10400, Thailand

A 31-year-old man presented with a worsening 6-month history of atraumatic lower back pain and tingling in the left leg. Digital rectal examination was unremarkable, and there were no signs of cauda equina syndrome clinically or of spinal cord compression on contrast computed tomography. Over the next 4 days he developed increasing weakness and hyporeflexia in the lower limbs bilaterally as well as urinary and fecal incontinence.

He was an Australian former missionary who had worked in Uganda for 2 months before returning to his home country 9 months prior to presentation. While in Uganda he had swum in Lake Victoria several times, shortly after one of which he had a self-limiting flu-like illness with symptoms of fever, headache, and malaise. He reported that some of his co-swimmers had also experienced similar acute symptoms and were later diagnosed with a “parasitic worm infection.”

**Figure 1.**
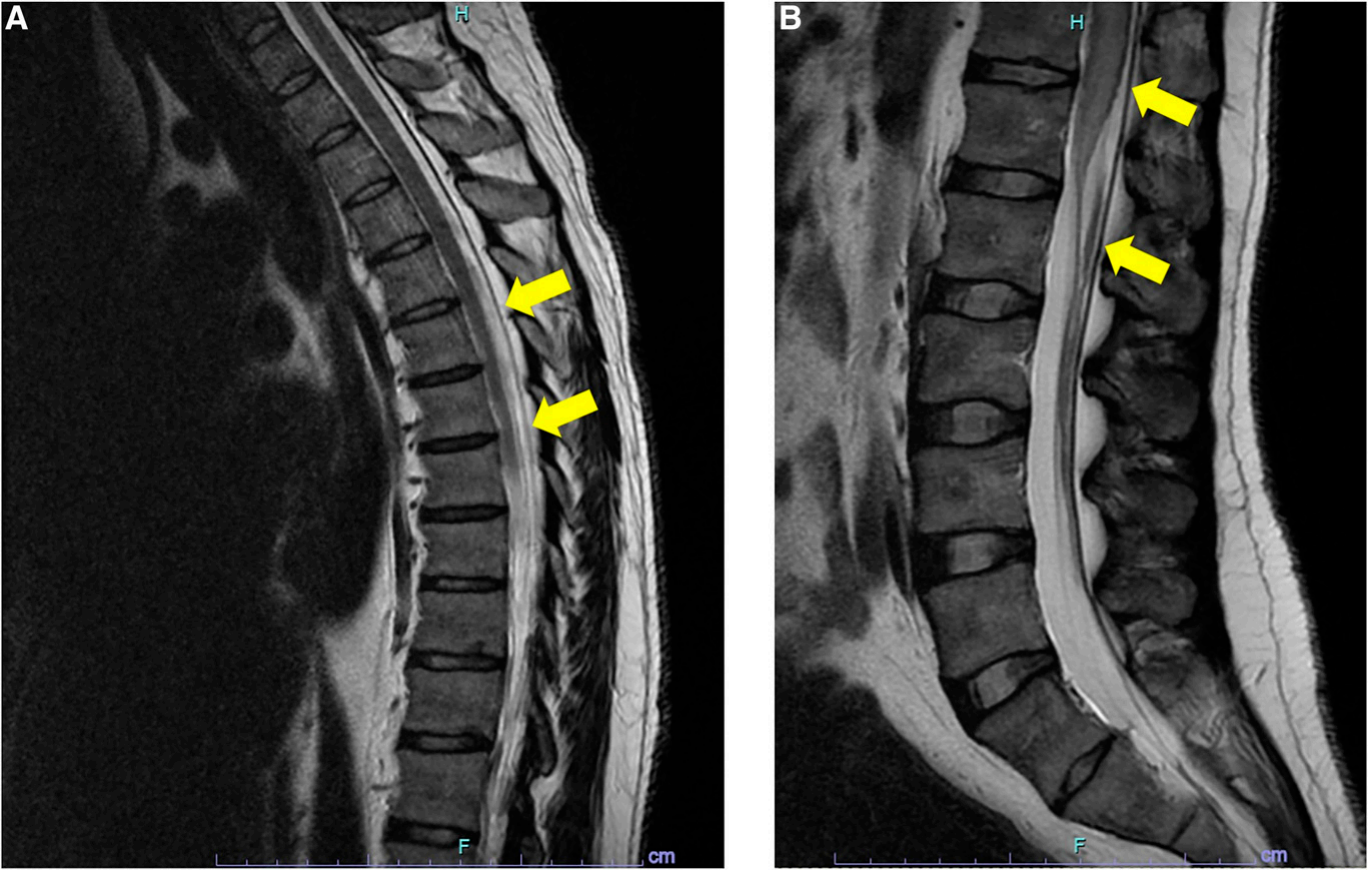
Initial MRI with contrast showing features of transverse myelitis. (**A**) Hyperintense T2 signal from T8 to the conus medullaris (arrows) and (**B**) edema of the conus medullaris with heterogenous enhancement post-contrast (arrows). The cause was probable neuroschistosomiasis, an inflammatory response to schistosomal ova deposited in the spinal cord. This figure appears in color at www.ajtmh.org.

Magnetic resonance imaging with contrast showed transverse myelitis, with hyperintense T2 signal from T8 to the conus medullaris, spinal cord edema, and heterogenous enhancement (Figure 1). He tested negative for anti-neuronal, anti-aquaporin-4, anti–glutamic acid decarboxylase, anti-nuclear, and anti-extractable nuclear antigen antibodies. Echinococcal, HIV, human T-lymphotropic virus I/II, Epstein-Barr virus, cytomegalovirus, Herpes simplex virus 1 and 2, and syphilis serology were also negative, as was an interferon-γ release assay for tuberculosis. Stool and urine microscopy were negative for *Schistosoma* ova. He declined rectal biopsy, but schistosomal IgG was strongly reactive in both blood and cerebrospinal fluid. He, thus, fulfilled the Brazilian national diagnostic criteria for probable spinal neuroschistosomiasis (clinical features of myelopathy, evidence of exposure to schistosomes [positive serology and previous symptoms consistent with acute schistosomiasis], evidence of an inflammatory spinal cord lesion on cerebrospinal fluid and imaging, and exclusion of other causes of transverse myelitis).^1^^,^^2^ The clinical features were also consistent with previously reported cases of spinal neuroschistosomiasis.^3^^,^^4^ It was not possible to identify precisely the causative species because species-specific serological testing was not available, but given his travel history and the non-endemicity of human pathogenic schistosomes to Australia, the most likely causative species was either *Schistosoma mansoni* or *Schistosoma haematobium*.

**Figure 2.**
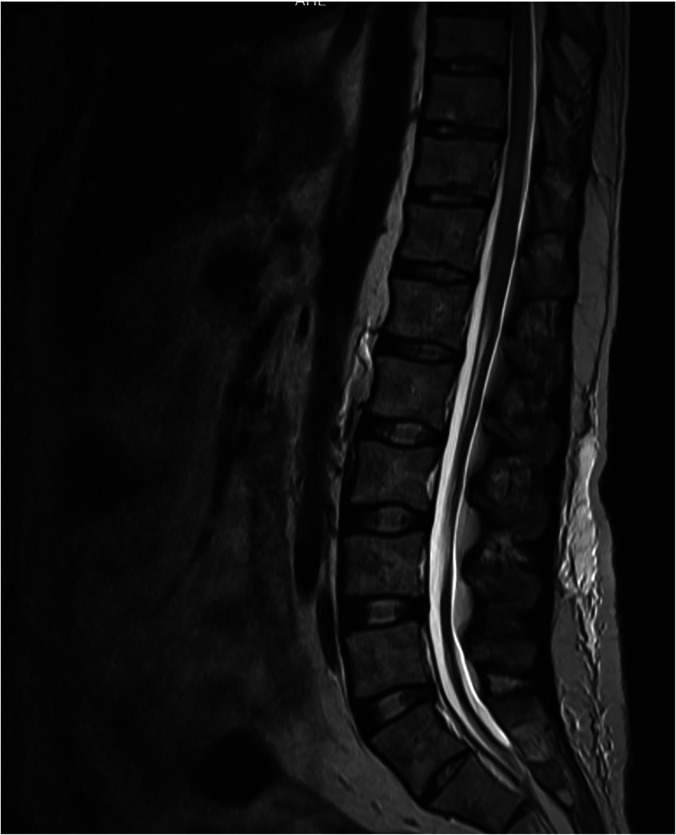
Progress MRI with contrast after 3 months of treatment of neuroschistosomiasis with praziquantel and pulsed methylprednisolone, followed by a tapering course of prednisolone. Compared with the initial MRI, the hyperintense T2 signal has decreased at T8–T12 and no longer affects the conus medullaris, and the edema of the conus medullaris has resolved.

He was commenced on treatment with praziquantel and pulsed methylprednisolone, followed by a slow tapering course of prednisolone.^4^^,^^5^ Despite radiological improvement 3 months after commencement of treatment (Figure 2), after 4 years he remained paraplegic and doubly incontinent with persisting neuropathic pain in the lower limbs. This rare cause of a rare neurological condition highlights the importance of a detailed travel and exposure history in the diagnostic process. This case also illustrates the variable prognosis of spinal neuroschistosomiasis.^4^^,^^5^
